# Growth differentiation factor-15/adiponectin ratio as a potential biomarker for metabolic syndrome in Han Chinese

**DOI:** 10.3389/fendo.2023.1146376

**Published:** 2023-04-19

**Authors:** Shuai Zheng, Min Shen, Yu Qian, Shushu Li, Yang Chen, Hemin Jiang, Hui Lv, Doudou Chen, Ruiling Zhao, Xuqin Zheng, Min Sun, Tao Yang, Yun Shi, Qi Fu

**Affiliations:** Department of Endocrinology and Metabolism, The First Affiliated Hospital of Nanjing Medical University, Nanjing, China

**Keywords:** GDF-15, adiponectin, GDF-15/adiponectin ratio, metabolic syndrome, biomarkers

## Abstract

**Aims:**

Growth differentiation factor-15 (GDF-15) and adiponectin are adipokines that regulate metabolism. This study aimed to evaluate the roles of GDF-15, adiponectin, and GDF-15/adiponectin ratio (G/A ratio) as biomarkers for detecting metabolic syndrome (MS).

**Materials and methods:**

This cross-sectional study included 676 participants aged 20–70 years in Jurong, China. The participants were divided into four groups based on sex and age (<40 and ≥40 years). MS was defined according to the modified National Cholesterol Education Program Adult Treatment Panel III criteria. Receiver operating characteristic curves were used to evaluate the performance of GDF-15, adiponectin, and the G/A ratio in predicting MS.

**Results:**

The prevalence of MS was 22.0% (149/676). Logistic regression analysis indicated that the G/A ratio and adiponectin levels, but not GDF-15 levels, were correlated with MS [odds ratio; 95% CI 1.010 (1.006–1.013) and 0.798 (0.735–0.865), respectively] after adjusting for confounding factors. The G/A ratio displayed a significant relationship with MS in each subgroup and with each MS component in both men and women; however, adiponectin concentrations were significantly associated with MS and all its components only in men (all *P <*0.05). The area under the curve (AUC) of the G/A ratio and the adiponectin level for MS was 0.758 and 0.748, respectively. The highest AUC was 0.757 for the adiponectin level in men and 0.724 for the G/A ratio in women.

**Conclusions:**

This study suggests that the G/A ratio and adiponectin are potential biomarkers for detecting MS in women and men, respectively.

## 1 Introduction

Metabolic syndrome (MS) is a cluster of coexisting metabolic risk factors, including central obesity, hyperglycemia, hypertension, and dyslipidemia, imposing significant burdens on global health (1). Therefore, there is an urgent need for appropriate and effective biomarkers to identify individuals at high risk of MS.

Growth differentiation factor 15 (GDF-15) belongs to the transforming growth factor (TGF)-β superfamily and is characterized by wide tissue distribution, anti-inflammatory effects, and its role in cellular responses to stress signals (2). Adipose tissue secretes GDF-15, which acts as an adipokine and may play a paracrine role in modulating adipose tissue function and body mass (3). Increased GDF-15 levels have been reported to be correlated with many metabolic diseases, including obesity, cardiovascular disease (CVD), type 2 diabetes mellitus (T2DM), and metabolic-associated fatty liver disease (4). A few studies have found that MS is associated with elevated GDF-15 levels among older adults (5–7). Therefore, both GDF-15 levels and MS prevalence are strongly affected by age (6, 8); however, the exact relationship between the two has not yet been determined.

Adiponectin is an adipocyte-specific factor that acts as a crucial bridge between adipose tissues and other metabolic organs (9). In addition, adiponectin reduces oxidative stress and inflammatory cytokines, thereby improving insulin resistance (IR) (10, 11). Previous cross-sectional studies have reported that hypoadiponectinemia is closely associated with MS and its components (12, 13); however, a meta-analysis has indicated that increased adiponectin level is an independent protector against the development of MS, especially in men (14). Moreover, the relationship between adiponectin and MS varies with sex (15) and race (16).

GDF-15 and adiponectin have been identified as metabolic coordinators, playing roles in improving lipolysis and IR through anti-inflammatory and antioxidative effects (2, 11). Higher GDF-15 concentrations and lower adiponectin levels are correlated with MS. Wu et al. first used the index of GDF-15/adiponectin ratio (G/A ratio), whose increment was independently associated with the risk of T2DM for all study populations, compared to GDF-15 or adiponectin alone, suggesting the combination of these two adipokines might have an “enhancing effect” on predicting the risk of T2DM (17). In this study, we aimed to evaluate the roles of GDF-15, adiponectin, and the G/A ratio as biomarkers for detecting MS.

## 2 Materials and methods

### 2.1 Study population

A total of 853 individuals aged 20–70 years living in Jurong City were recruited for this study. All subjects were ethnic Han Chinese. The final analysis was performed on 676 participants after excluding 107 participants treated with antidiabetic, antihypertensive, or lipid-lowering medications; 36 participants without GDF-15 measurements; 16 participants with CVD (e.g., myocardial infarction, stroke, or heart failure), autoimmune diseases, or cancers; and 18 participants with incomplete data. Based on age (40 years, which is the criterion used to divide young and middle-aged individuals) and sex, the cohort was divided into four groups: group 1 (age <40 years, women, n=185), group 2 (age <40 years, men, n=175), group 3 (age ≥40 years, women, n=113), and group 4 (age ≥40 years, men, n=203). All participants provided written informed consent, and the study was approved by the institutional review board of the First Affiliated Hospital of Nanjing Medical University (2021-SR-298).

### 2.2 Data collection and measurements

The participants completed a detailed health and lifestyle questionnaire, which included questions on age, sex, smoking and drinking status, physical activity, education, disease history, and medication information. Smoking status was divided into three categories: current, former, and never-smoker. Current drinking was referred to as “alcohol consumption at least once per week for the previous 6 months”. Regular exercise was defined as engaging in 30 min of leisure-time activities at least three times a week, including jogging, swimming, cycling, playing ball, dancing, and mountain climbing. Education level was classified as primary school or below, middle or high school, or college or above.

After a 12–h overnight fasting, the participants underwent anthropometric evaluation and blood collection. The separated plasma was stored at −80°C before analysis. Measurements included height, weight, and waist circumference (WC) according to a standard protocol. Body mass index (BMI) was calculated as the weight (kg)/height squared (m^2^). Blood pressure (BP) was measured twice with 1–2 min intervals to obtain average readings after at least 5 min rest.

Serum GDF-15 concentrations were measured using a Human Quantikine ELISA Kit (R&D Systems, Minneapolis, MN, USA). Adiponectin and insulin levels were detected using chemiluminescence detection kits (YHLO Biotech, Shenzhen, China). Fasting plasma glucose (FPG) levels were measured using the hexokinase method (AU5400; Olympus, Tokyo, Japan). Glycated hemoglobin (HbA1c) levels were examined using capillary blood samples (Variant II; Bio-Rad, Hercules, USA). Levels of triglycerides (TG), low-density lipoprotein cholesterol (LDL-C), high-density lipoprotein cholesterol (HDL-C), and total cholesterol (TC) were measured using a chemiluminescence autoanalyzer (Modular E170; Roche, Basel, Switzerland). HOMA-IR was calculated as follows: FPG (mmol/L) × fasting insulin (mIU/L)/22.5 (18).

### 2.3 Definition of MS

MS was defined as the presence of at least three of the following abnormalities according to the modified NCEP ATP III criteria for Asians (19): (1) abdominal obesity: WC ≥90 cm in men or ≥80 cm in women; (2) hypertension: systolic BP ≥130 mmHg or diastolic BP ≥85 mmHg or drug-treated hypertension; (3) TG ≥150 mg/dL (1.70 mmol/L) or drug treatment for elevated TG; (4) HDL-C <40 mg/dL (1.03 mmol/L) for men or <50 mg/dL (1.30 mmol/L) for women or drug treatment for low HDL-C; and (5) hyperglycemia: FPG ≥110 mg/dL (6.1 mmol/L) or taking hypoglycaemic agents.

### 2.4 Statistical analysis

Statistical analyses were conducted using SPSS 23.0. Data are presented as the mean ± SD, number (%), or median (interquartile range). For continuous variables, Student’s t-test or Mann–Whitney U test was performed between groups. Categorical variables were compared using X^2^ tests. Correlations between various indicators of metabolic parameters were evaluated using Spearman’s correlation. A logistic regression model was used to determine the independent effects of GDF-15 levels, adiponectin levels, and the G/A ratio on MS and its components. Restricted cubic spline regression was used to assess dose-response relationships. Potential confounding factors were included in the model to minimize bias. Receiver operating characteristic (ROC) curves were drawn to evaluate the performance of the potential biomarkers in identifying participants with MS. A two-sided *P <*0.05 was considered statistically significant.

## 3 Results

### 3.1 Baseline characteristics

Among the 676 individuals (378 men and 298 women) included in the final analysis, the prevalence of MS was 22.0% (149/676). Individuals with MS were mainly older, less-educated men, with a higher rate of smoking and drinking, compared with those without MS. No significant difference in regular exercise was observed between the two groups. GDF-15 levels and the G/A ratio were significantly higher in patients with MS, whereas the adiponectin level was considerably lower than that in participants without MS. Demographic and metabolic characteristics were further analyzed according to sex. These results indicated that men had higher levels of WC, BP, TG, FPG, GDF-15, and the G/A ratio but lower levels of HDL and adiponectin than women (**Table 1**).

**Table 1:** Baseline characteristics of the study population according to metabolic status and sex.

	Metabolic Status			Sex		
	Metabolic syndrome	No metabolic syndrome	*P* value	Men	Women	*P* value
	(n=149)	(n=527)		(n=378)	(n=298)	
Sex, men (%)	114 (76.5)	264 (50.1)	< 0.001			
Age (years)	44.6 (10.2)	38.8 (11.6)	< 0.001	41.9 (12.1)	37.8 (10.5)	< 0.001
BMI, kg/m^2^	28.2 (3.0)	23.3 (3.0)	< 0.001	25.4 (3.6)	23.0 (3.1)	< 0.001
WC, cm	94.2 (7.5)	79.8 (8.9)	< 0.001	88.0 (9.2)	76.6 (8.3)	< 0.001
SBP, mmHg	135.1 (15.0)	119.6 (13.6)	< 0.001	129.0 (13.4)	115.4 (14.2)	< 0.001
DBP, mmHg	91.1 (8.9)	80.5 (9.4)	< 0.001	86.5 (9.6)	78.1 (9.1)	< 0.001
TG, mmol/L	2.3 (1.6, 3.0)	0.9 (0.7, 1.3)	< 0.001	1.3 (0.9, 2.0)	0.9 (0.7, 1.3)	< 0.001
LDL-C, mmol/L	3.4 (0.7)	3.0 (0.8)	< 0.001	3.2 (0.8)	2.9 (0.8)	< 0.001
HDL-C, mmol/L	1.2 (0.3)	1.6 (0.3)	< 0.001	1.4 (0.3)	1.6 (0.3)	< 0.001
TC, mmol/L	5.2 (0.9)	4.7 (0.9)	< 0.001	4.9 (0.9)	4.7 (0.9)	0.028
FPG, mmol/L	6.5 (1.4)	5.7 (0.6)	< 0.001	6.0 (1.1)	5.6 (0.6)	< 0.001
HbA1c, % HbA1c, mmol/mol	5.9 (0.8) 40.6 (9.3)	5.4 (0.4) 35.2 (4.4)	< 0.001 < 0.001	5.6 (0.7) 37.3 (7.5)	5.4 (0.4) 35.2 (3.9)	< 0.001 < 0.001
HOMA-IR	4.5 (3.5, 6.4)	2.8 (2.1, 3.6)	< 0.001	3.2 (2.3, 4.6)	2.8 (2.1, 3.7)	0.001
GDF-15, pg/ml	739.3 (542.0, 938.6)	585.5 (454.8, 794.2)	< 0.001	695.9 (514.2, 937.8)	557.4 (439.6, 719.8)	< 0.001
Adiponectin, mg/L	5.4 (4.2, 7.1)	8.0 (6.3, 10.5)	< 0.001	6.9 (5.3, 8.9)	8.3 (6.2, 10.8)	< 0.001
GDF-15/adiponectin, 10^-6^	122.8 (90.8, 193.2)	74.0 (49.7, 111.7)	< 0.001	100.2 (63.6, 158.2)	69.9 (48.3, 100.2)	< 0.001
Smoking, n (%)			< 0.001			< 0.001
Current	77 (51.7)	130 (24.6)		206 (54.5)	1 (0.3)	
Former	11 (7.4)	15 (2.8)		25 (6.6)	1 (0.3)	
Never	61 (40.9)	382 (72.5)		147 (38.9)	296 (99.3)	
Current drinking, n (%)	51 (34.2)	76 (14.4)	< 0.001	123 (32.5)	4 (1.3)	< 0.001
Regular exercise, n (%)	63 (42.3)	187 (35.5)	0.129	156 (41.3)	94 (31.5)	0.009
Education, n (%)			< 0.001			< 0.001
Primary school or below	11 (7.4)	23 (4.4)		15 (4.0)	19 (6.4)	
Middle or high school	89 (59.7)	210 (39.8)		192 (50.8)	107 (35.9)	
College or above	49 (32.9)	294 (55.8)		171 (45.2)	172 (57.7)	

Data are presented as mean ± SD, numbers (%), or median (interquartile ranges). BMI, body mass index; WC, waist circumference; SBP, systolic blood pressure; DBP, diastolic blood pressure; TG, triglycerides; LDL-C, low-density lipoprotein cholesterol; HDL-C, high-density lipoprotein cholesterol; TC, total cholesterol; FPG, fasting plasma glucose; HbA1c, glycated hemoglobin; HOMA-IR, homeostasis model assessment -insulin resistance; GDF-15, growth differentiation factor 15.

### 3.2 Spearman correlations of GDF-15, adiponectin, and G/A ratio with metabolic parameters

The components of MS (WC, BP, TG, FPG, and HDL) were significantly correlated with levels of GDF-15, adiponectin, and the G/A ratio (all *P <*0.01). HOMA-IR was positively correlated with the G/A ratio and negatively correlated with adiponectin concentrations, but not with GDF-15 concentrations (**Supplementary Table S1**).

### 3.3 Association between MS and GDF-15, adiponectin, and G/A ratio

Logistic regression analysis showed that MS was significantly associated with a lower level of adiponectin and a higher G/A ratio, but not with the GDF-15 level, after adjusting for age and sex. Model 2 was further adjusted for multiple factors (e.g., smoking, drinking, regular exercise, and education level), and the results showed that the G/A ratio and adiponectin levels were significantly correlated with MS with odds ratios (ORs) and a 95% confidence interval (CI) of 1.010 (1.006–1.013) and 0.798 (0.735–0.865), respectively (**Table 2**). Adjusted ORs for MS according to the quartiles of the G/A ratio or adiponectin levels were shown in **Supplementary Table S2**.

**Table 2:** Odds ratios for metabolic syndrome according to GDF-15, adiponectin, and GDF-15/adiponectin.

**Total participants (n=676)**			GDF-15	Adiponectin	GDF-15/adiponectin
		Model 1	1.000 (0.999−1.001)	0.794 (0.733−0.861)	1.010 (1.007−1.013)
			*P* = 0.815	*P* < 0.001	*P* < 0.001
		Model 2	1.000 (0.999−1.001)	0.798 (0.735−0.865)	1.010 (1.006−1.013)
			*P* = 0.968	*P* < 0.001	*P* < 0.001
**Subgroups stratified by age and sex (n=676)**	Group 1 (n=185)	Model 3	1.001 (0.998−1.004)	0.901 (0.746−1.088)	1.019 (1.007−1.030)
			*P* = 0.503	*P* = 0.278	*P* = 0.002
		Model 4	1.001 (0.998−1.004)	0.932 (0.782−1.109)	1.017 (1.005−1.030)
			*P* = 0.611	*P* = 0.425	*P* = 0.004
	Group 2 (n=175)	Model 3	1.001 (1.000−1.003)	0.386 (0.273−0.548)	1.017 (1.010−1.024)
			*P* = 0.054	*P* < 0.001	*P* < 0.001
		Model 4	1.001 (0.999−1.003)	0.407 (0.285−0.582)	1.015 (1.008−1.022)
			*P* = 0.290	*P* < 0.001	*P* < 0.001
	Group 3 (n=113)	Model 3	1.000 (0.998−1.002)	0.852 (0.717−1.012)	1.014 (1.003−1.024)
			*P* = 0.676	*P* = 0.068	*P* = 0.011
		Model 4	1.000 (0.998−1.003)	0.833 (0.693−1.002)	1.017 (1.004−1.030)
			*P* = 0.763	*P* = 0.053	*P* = 0.009
	Group 4 (n=203)	Model 3	1.000 (0.999−1.001)	0.882 (0.797−0.977)	1.005 (1.001−1.009)
			*P* = 0.429	*P* = 0.016	*P* = 0.015
		Model 4	1.000 (0.999−1.001)	0.886 (0.799−0.981)	1.006 (1.002−1.010)
			*P* = 0.979	*P* = 0.020	*P* = 0.006

Model 1, adjusted for age and sex; Model 2, Model 1 plus adjusted for smoking, drinking, regular exercise, and education level; Model 3, unadjusted; Model 4, adjusted for age, smoking, drinking, regular exercise, and education level. Group 1, age <40 years, women; Group 2, age <40 years, men; Group 3, age ≥40 years, women; Group 4, age ≥40 years, men.

Subgroup analyses were performed according to age and sex. The prevalence of MS in groups 1, 2, 3, and 4 was 7.6%, 20.6%, 18.6%, and 38.4%, respectively. MS was significantly associated with a higher G/A ratio in all four groups; however, the relationship between MS and adiponectin levels was only significant in groups 2 and 4. Significant associations were retained after adjusting for age and other multiple factors (**Table 2**).

**Figure 1:**
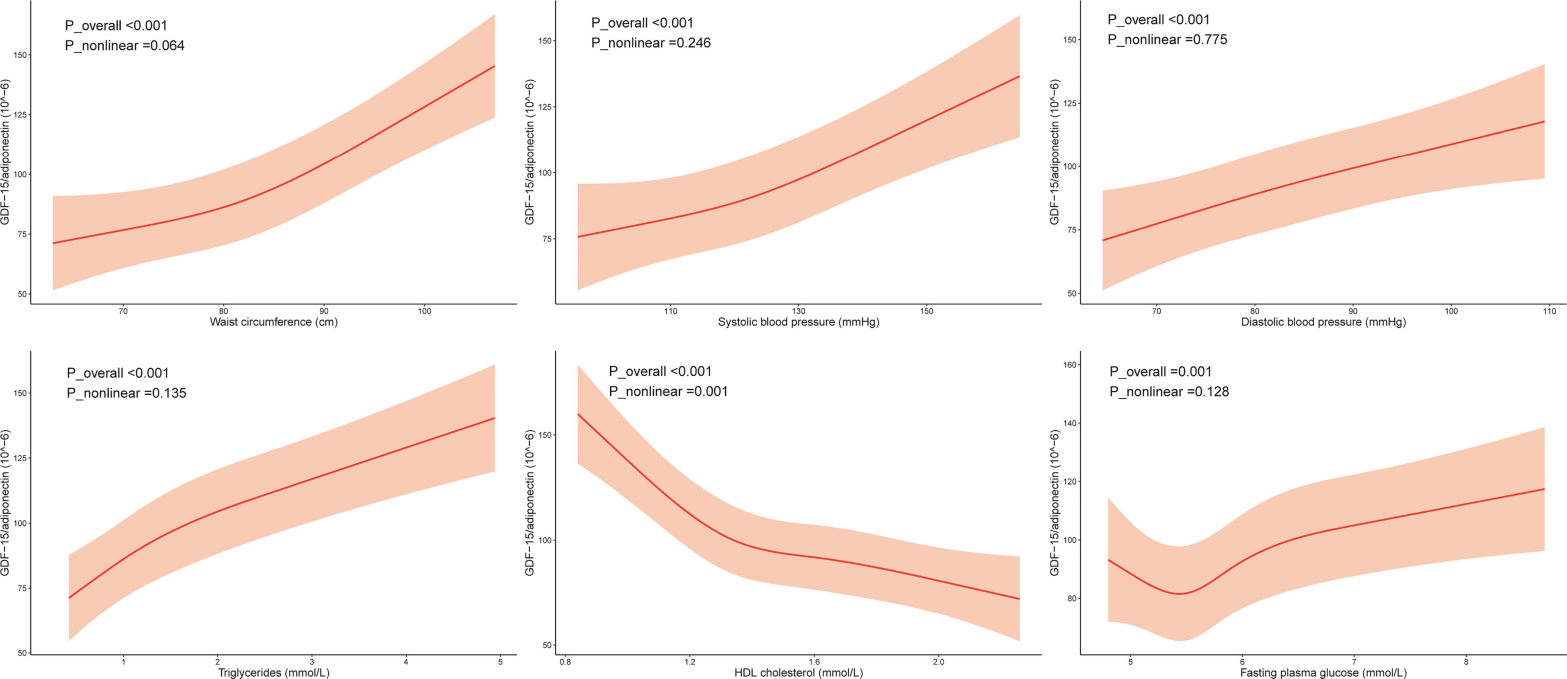
Association of the components of metabolic syndrome with GDF-15/adiponectin. Restricted cubic splines (RCS) were used, and the minimum AIC value was chosen as the optimal number of knots. The model was adjusted for age, sex, smoking, drinking, regular exercise, and education level. The RCS knots were located at 75.0–82.0–90.3 cm for waist circumference, 112.5–121.5–132.0 mm Hg for systolic blood pressure, 75.5–82.0–89.5 mm Hg for diastolic blood pressure, 0.7–1.1–1.6 mmol/L for triglyceride levels, 1.2–1.4–1.6–1.8 for HDL cholesterol levels, and 5.3–5.6–5.8–6.2 mmol/L for fasting plasma glucose.

**Table 3** shows the ORs for MS components based on adiponectin levels and the G/A ratio. Adiponectin levels and the G/A ratio were correlated with each MS component after adjusting for confounding factors. Sex-stratified analysis was conducted, and the results showed that the G/A ratio was associated with each MS component in both men and women; nevertheless, the adiponectin concentration was not correlated with high BP in women but had a relatively strong correlation with high TG levels in men. The GDF-15 level was not associated with any MS component (**Supplementary Table S3**). Additionally, we observed a dose-response relationship between MS components and the G/A ratio and adiponectin levels (**Figure 1**; **Supplementary Figure S1**).

**Table 3:** Adjusted odds ratios for the components of metabolic syndrome according to adiponectin and GDF-15/adiponectin.

	Adiponectin			GDF-15/adiponectin		
	Total	Men	Women	Total	Men	Women
High waist circumference ^a^	0.910 (0.864−0.959) *P* < 0.001	0.910 (0.850−0.974) *P* =0.007	0.910 (0.836−0.990) *P* =0.028	1.007 (1.004−1.010) *P* < 0.001	1.006 (1.003−1.010) *P* < 0.001	1.010 (1.003−1.016) *P* = 0.003
High blood pressure ^b^	0.925 (0.880−0.973) *P* =0.003	0.919 (0.863−0.978) *P* =0.008	0.923 (0.841−1.014) *P* =0.094	1.007 (1.003−1.010) *P* < 0.001	1.007 (1.003−1.011) *P* < 0.001	1.008 (1.001−1.015) *P* = 0.021
High TG ^c^	0.874 (0.817−0.935) *P* < 0.001	0.569 (0.454−0.713) *P* < 0.001	0.833 (0.731−0.951) *P* =0.007	1.007 (1.004−1.010) *P* < 0.001	1.010 (1.005−1.014) *P* < 0.001	1.013 (1.005−1.021) *P* = 0.001
Low HDL cholesterol ^d^	0.771 (0.694−0.855) *P* < 0.001	0.771 (0.694−0.855) *P* < 0.001	0.874 (0.778−0.981) *P* =0.023	1.010 (1.006−1.014) *P* < 0.001	1.005 (1.001−1.008) *P* = 0.006	1.012 (1.005−1.020) *P* = 0.001
High blood glucose ^e^	0.873 (0.818−0.932) *P* < 0.001	0.859 (0.790−0.934) *P* < 0.001	0.877 (0.781−0.985) *P* =0.026	1.005 (1.002−1.008) *P* = 0.001	1.005 (1.002−1.008) *P* = 0.001	1.007 (1.000−1.015) *P* = 0.047

Adjusted for age, sex, smoking, drinking, regular exercise, and education level. ^a^ waist circumference ≥90 cm in men or ≥80 cm in women; ^b^ systolic blood pressure ≥130 mmHg or diastolic blood pressure ≥85 mmHg; ^c^ TG ≥150 mg/dL; ^d^ HDL-C <40 mg/dL for men or <50 mg/dL for women; ^e^ fasting blood glucose ≥110 mg/dL.

### 3.4 ROC curve for the detection of MS

**Figure 2:**
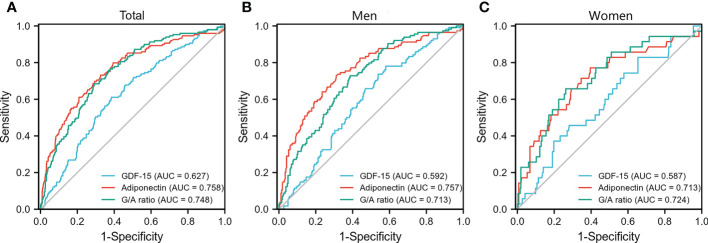
Receiver operating characteristic curve for the detection of metabolic syndrome. Receiver operating characteristic curves of GDF-15, adiponectin, and G/A ratio for the detection of metabolic syndrome in the total population **(A)**, men **(B)**, and women **(C)**, respectively. G/A ratio, GDF-15/adiponectin ratio.

ROC analysis was performed to compare the predictive power of GDF-15, adiponectin, and the G/A ratio for MS. In the total population, the area under the curve (AUC) of GDF-15 levels, adiponectin levels, and the G/A ratio was 0.627, 0.758, and 0.748, respectively (**Figure 2A**). The highest AUC was 0.757 for the adiponectin level in men and 0.724 for the G/A ratio in women (**Figures 2B, C**).

## 4 Discussion

In this study, we found that the G/A ratio and adiponectin, but not GDF-15, were significantly associated with MS and its components in all participants aged 20–70 years after controlling for confounding variables. Furthermore, the G/A ratio displayed a significant relationship with MS in each subgroup stratified by sex and age (40 years) and was correlated with each component of MS in both men and women. Nevertheless, adiponectin showed a better diagnostic performance in classifying men with MS. This finding implies that the G/A ratio and adiponectin are potential biomarkers for detecting MS in women and men, respectively.

Recent studies have found that GDF-15 regulates lipid and carbohydrate metabolism, reduces food intake and body mass, and improves insulin sensitivity (20, 21), suggesting its potential therapeutic applications in metabolic diseases. GDF-15 levels in ob/ob mice and obese high-fat-fed mice were significantly higher than in the control (22). Further treatment with recombinant GDF-15 in obese mice significantly reduced body weight, whereas the use of GDF-15 antibody significantly increased body weight (22, 23). In addition, GDF-15 knockout mice on high-fat diets were more prone to obesity (24). Therefore, GDF-15 could be a compensatory protective factor in obesity. Similar results to animal tests, GDF-15 levels are correlated with obesity and also positively associated with adipose tissue mass and body weight after correcting for age and sex (25, 26). However, few studies have directly explored the association between GDF-15 and MS. Adrián et al. found that MS was associated with elevated GDF-15 levels in participants aged ≥65 years. High WC, increased FPG, and low HDL-C levels were the main drivers of this association (5). Two other studies in older participants (average age: 80 and 59 years) also showed that elevated GDF-15 levels were associated with MS (6, 7). Among younger individuals, a case-control study of 40 participants with obesity (mean age: 34 years) reported higher GDF-15 levels in patients with MS than in the control group without MS; nevertheless, GDF-15 concentration was not associated with any component of MS (27). Ho et al. found GDF-15 levels were positively related to the presence of MS, after recruiting 279 subjects younger than 65 years old. When substituting MS with its components, only hyperglycemia was positively correlated with GDF-15 levels (28). In the present study, GDF-15 levels were not associated with MS and its components in all participants aged 20–70 years and subgroups stratified by age and sex. This observation could be because of age, as GDF-15 levels are highly influenced by age, or because of other factors, including differences in obesity and sample size.

Adiponectin levels are reduced in participants with obesity, and this reduction is proposed to play a crucial role in the pathogenesis of CVD associated with obesity and MS (29). Additionally, adiponectin is associated with MS regardless of age (13, 30); however, this association varies with sex and race. Adiponectin concentration is reported to be lower in men than in women, partly because of the ability of androgens to suppress adiponectin (31), which might contribute to severe IR in men (29). Prospective studies on the Korean population have reported that adiponectin levels improve the clinical prediction of MS in men but not in women (32, 33). However, a prospective study on Japanese-Americans suggested that low levels of total and high-molecular-weight adiponectin might be a possible predictor of MS in both men and women (34). Previous studies have shown that Asian populations have lower adiponectin concentrations than Caucasians (35, 36). Moreover, adiponectin is correlated with MS in Chinese and Caucasian populations (12, 13), but not in the Indonesian population (16). Additionally, the relationship between adiponectin and each MS component varies among participants of different races and sexes (12, 29). In the present study, an inverse association was found between adiponectin levels and MS in the total population and men, but not in women, irrespective of age. ROC curves showed that adiponectin was a preferential marker for MS in men compared to GDF-15 or the G/A ratio. Furthermore, the adiponectin level was correlated with each MS component in the total population and the sex-stratified subgroups, except for BP in women. However, the high prevalence of MS in men might amplify the effect of adiponectin in detecting MS. Therefore, the effects of sex and race on the association between adiponectin and MS and its components need further investigation.

Obesity-related IR is central to the development of MS. Both GDF-15 and adiponectin have a protective role in regulating insulin sensitivity, weight gain, and inflammation (4, 12). Although GDF-15 is expressed in various tissues, its role as an adipokine that regulates metabolism, which overlaps with the functions of adiponectin, has attracted increasing attention. In addition, GDF-15 and adiponectin can improve IR *via* the activation of adenosine monophosphate-activated protein kinase (AMPK), a cellular energy sensor, in the liver and skeletal muscles, respectively (37, 38). The G/A ratio is a recently proposed index whose increment was independently correlated with the risk of T2DM in all study populations aged 18–70 years and subgroups, compared to GDF-15 or adiponectin levels alone after adjusting for confounders; however, adiponectin concentrations had a stronger association with T2DM in relatively healthy men, but not in relatively healthy women and participants with metabolic disorders (17). In the present study, we used the G/A ratio to assess the ORs of MS and its components and found that the G/A ratio was associated with MS in all participants and age- and sex-stratified subgroups, correlating with each MS component in both men and women. Furthermore, a significant dose-response relationship was observed between the G/A ratio and each MS component. Our results were consistent with those of Wu et al., showing that adiponectin is a biomarker for evaluating T2DM and MS in men, whereas the G/A ratio benefited both men and women, suggesting that the combination of these two adipokines may have an “enhancing effect”. Additionally, previous studies have explored the association between GDF-15 and adiponectin. Tsai et al. found that GDF-15 treatment reduced adiposity and corrected metabolic dysfunction in high-fat diet-fed mice, accompanied by higher circulating adiponectin levels (39). Recombinant GDF-15 enhanced adiponectin release from adipocytes (3), and GDF-15-overexpressing transgenic mice showed upregulated adiponectin at the mRNA level (40), implying that GDF-15 is a positive regulator of adiponectin (3). In an obesity-related disease state, higher GDF-15 concentrations and lower adiponectin levels have been observed (41). The obesity-related disease is a chronic inflammatory status, in which the compensatory protective effect of elevated GDF-15 might be due to its anti-inflammatory role, but eventually futile (42). We speculate that a compensatory increase of GDF-15 levels improves adiponectin concentration; however, the elevated level of GDF-15 is limited, and it cannot offset the decrease in adiponectin level that accompanies the chronic low-grade inflammatory progression of the disease. The G/A ratio can potentially exclude confounding factors, such as age, sex, body composition, and several life habits that can be correlated with MS and its components. However, further studies are required to elucidate this mechanism.

Our study has several advantages. First, our analysis adjusted for several confounding factors, such as smoking, drinking, regular exercise, and education level, in addition to age and sex. We also excluded participants with hypoglycaemic, antihypertensive, and lipid-lowering drugs and some disease states, such as CVD, autoimmune diseases, and cancers, to avoid the impact on the detection of GDF-15 and adiponectin. In addition, restricted cubic splines were used to reflect the dose-response relationship between biomarkers and MS on a continuous scale. However, this study also has several limitations. First, because of the cross-sectional data, we could not determine whether the G/A ratio plays a causal role in the pathogenesis of MS. Second, accurately determining the relationship between the G/A ratio and MS was challenging, given the relatively small sample sizes. Finally, our study was conducted only in an Eastern Chinese Han population; therefore, it is unclear whether our results could be generalized to different ethnic groups.

In conclusion, our study indicates a significant and independent association between an increased G/A ratio and MS and each of its components in the total study population and all subgroups. Our results indicate that the G/A ratio and adiponectin are potential biomarkers for detecting MS in women and men, respectively. Future prospective studies should confirm these findings in a larger number of participants across different age groups, sexes, and races. In addition, the relationship between GDF-15 and adiponectin needs to be comprehensively explored.

## Data availability statement

The original contributions presented in the study are included in the article/**Supplementary Material**. Further inquiries can be directed to the corresponding authors.

## Ethics statement

The studies involving human participants were reviewed and approved by the institutional review board of the First Affiliated Hospital of Nanjing Medical University. The patients/participants provided their written informed consent to participate in this study.

## Author contributions

SZ analyzed the data and wrote the manuscript; MShen and YQ made data analyses and performed experimental work; SL, YC, HJ, HL, DC, and RZ made data collection; XZ, MSun, and TY provided valuable advice; QF and YS designed the study and reviewed the article. All authors have read and approved the submitted version.
