# Supplementary material for: Growth differentiation factor-15/adiponectin ratio as a potential biomarker for metabolic syndrome in Han Chinese
Source: Front Endocrinol (Lausanne). 2023 Apr 19;14:1146376. doi: 10.3389/fendo.2023.1146376 (PMC10154592; doi:10.3389/fendo.2023.1146376)
Supplement: Supplementary file 1 [file Image_1.pdf]

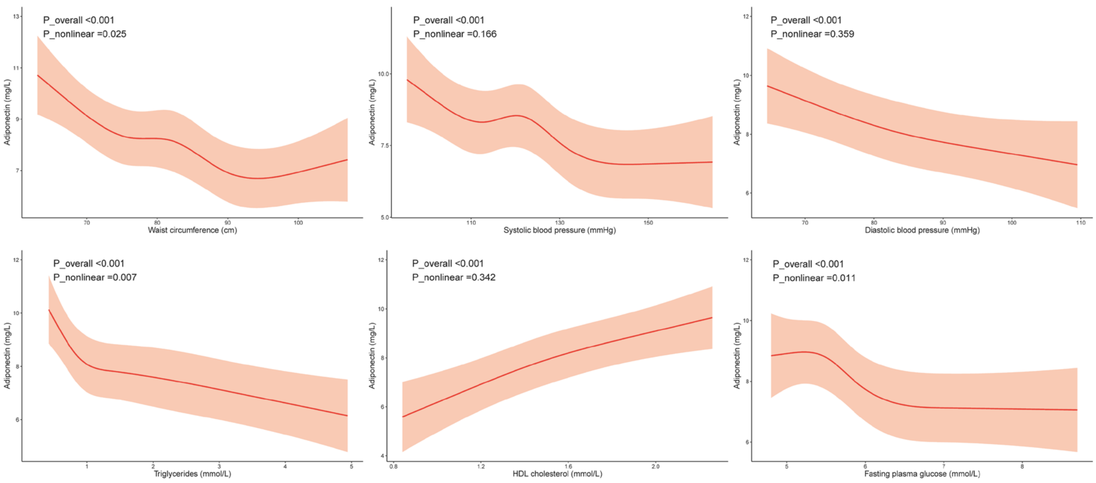

Fig. S1 Association of the components of metabolic syndrome with adiponectin

Restricted cubic splines (RCS) were used, and the minimum AIC value was chosen as the optimal number of knots. The model was adjusted for age, sex, smoking, drinking, regular exercise, and education level. The RCS knots were located at 72.9–78.0–82.0–87.8–94.0–117.0 cm for waist circumference, 109.5–114.9–121.5–128.0–137.0–201.0 mm Hg for systolic blood pressure, 75.5–82.0–89.5 mm Hg for diastolic blood pressure, 0.7–0.9–1.3–1.9 mmol/L for triglyceride levels, 1.3–1.5–1.7 for HDL cholesterol levels, and 5.3–5.6–5.8–6.2 mmol/L for fasting plasma glucose.
