# Supplementary material for: Growth differentiation factor-15/adiponectin ratio as a potential biomarker for metabolic syndrome in Han Chinese
Source: Front Endocrinol (Lausanne). 2023 Apr 19;14:1146376. doi: 10.3389/fendo.2023.1146376 (PMC10154592; doi:10.3389/fendo.2023.1146376)
Supplement: Supplementary file 2 [file Table_1.pdf]

Table S1 Spearman correlation between GDF-15, adiponectin, and GDF-15/ adiponectin with metabolic parameters

	GDF-15		Adiponectin		GDF-15/ adiponectin	
	r	*p*	r	*p*	r	*p*
Age	0.482	< 0.001	−0.057	0.141	0.364	< 0.001
BMI	0.160	< 0.001	−0.401	< 0.001	0.353	< 0.001
WC	0.270	< 0.001	−0.380	< 0.001	0.417	< 0.001
SBP	0.265	< 0.001	−0.264	< 0.001	0.353	< 0.001
DBP	0.228	< 0.001	−0.268	< 0.001	0.327	< 0.001
TG	0.138	< 0.001	−0.417	< 0.001	0.351	< 0.001
LDL-C	0.122	0.002	−0.158	< 0.001	0.183	< 0.001
HDL-C	−0.118	0.002	0.408	< 0.001	-0.337	< 0.001
TC	0.147	< 0.001	−0.066	0.088	0.141	< 0.001
FPG	0.161	< 0.001	−0.263	< 0.001	0.283	< 0.001
HbA1c	0.259	< 0.001	−0.220	<0.001	0.307	< 0.001
HOMA-IR	−0.052	0.180	−0.249	<0.001	0.113	0.003
GDF-15			−0.114	0.003	0.732	< 0.001

BMI, body mass index; WC, waist circumference; SBP, systolic blood pressure; DBP, diastolic blood pressure; TG, triglycerides; LDL-C, low-density lipoprotein cholesterol; HDL-C, high-density lipoprotein cholesterol; TC, total cholesterol; FPG, fasting plasma glucose; HbA1c, glycated hemoglobin; HOMA-IR, homeostasis model assessment -insulin resistance.
