# Supplementary material for: Growth differentiation factor-15/adiponectin ratio as a potential biomarker for metabolic syndrome in Han Chinese
Source: Front Endocrinol (Lausanne). 2023 Apr 19;14:1146376. doi: 10.3389/fendo.2023.1146376 (PMC10154592; doi:10.3389/fendo.2023.1146376)
Supplement: Supplementary file 3 [file Table_2.pdf]

Table S2 Adjusted odds ratios of metabolic syndrome according to quartiles of GDF-15, adiponectin, and GDF-15/ adiponectin

	Q1	Q2	Q3	Q4	Trend *p*
GDF-15	1.00	1.640 (0.868−3.099)	1.767 (0.942−3.314)	1.751 (0.909−3.373)	0.295
Adiponectin	1.00	0.328 (0.198−0.544)	0.104 (0.055−0.196)	0.096 (0.048−0.191)	< 0.001
G/A ratio	1.00	2.621 (1.153−5.955)	5.038 (2.300−11.038)	7.909 (3.607−17.341)	< 0.001

Adjusted for age, sex, smoking, drinking, regular exercise, and education level. G/A ratio, GDF-15/ adiponectin ratio.
