# Supplementary material for: Growth differentiation factor-15/adiponectin ratio as a potential biomarker for metabolic syndrome in Han Chinese
Source: Front Endocrinol (Lausanne). 2023 Apr 19;14:1146376. doi: 10.3389/fendo.2023.1146376 (PMC10154592; doi:10.3389/fendo.2023.1146376)
Supplement: Supplementary file 4 [file Table_3.pdf]

Table S3 Adjusted odds ratios for the components of metabolic syndrome according to GDF-15

	GDF-15		
	Total	Men	Women
High waist circumference [a]	1.000 (0.999−1.001)	1.000 (0.999−1.001)	1.000 (0.999−1.001)
	P = 0.963	*P* = 0.627	*P* = 0.922
High blood pressure [b]	1.001 (1.000−1.001)	1.001 (1.000−1.002)	1.000 (0.999−1.002)
	P =0.078	*P* = 0.064	*P* = 0.562
High TG [c]	1.000 (0.999−1.000)	1.000 (0.999−1.001)	0.999 (0.997−1.001)
	P = 0.314	*P* = 0.873	*P* = 0.166
Low HDL cholesterol [d]	1.000 (1.000−1.001)	1.001 (0.999−1.002)	1.000 (0.999−1.002)
	P = 0.359	*P* = 0.296	*P* = 0.842
High blood glucose [e]	1.000 (0.999−1.001)	1.000(0.999−1.001)	0.999 (0.997−1.001)
	P = 0.796	*P* = 0.855	*P* = 0.224

Adjusted for age, sex, smoking, drinking, regular exercise, and education level. [a] waist circumference ≥90 cm in men or ≥80 cm in women; [b] systolic blood pressure ≥130 mmHg or diastolic blood pressure ≥85 mmHg; [c] TG ≥150 mg/dL; [d] HDL-C <40 mg/dL for men or <50 mg/dL for women; [e] fasting blood glucose ≥110 mg/dL.
